# Pulse-Controlled Amplification–A new powerful tool for on-site diagnostics under resource limited conditions

**DOI:** 10.1371/journal.pntd.0009114

**Published:** 2021-01-29

**Authors:** Katharina Müller, Sarah Daßen, Scott Holowachuk, Katrin Zwirglmaier, Joachim Stehr, Federico Buersgens, Lars Ullerich, Kilian Stoecker

**Affiliations:** 1 Bundeswehr Institute of Microbiology, Munich, Germany; 2 GNA Biosolutions GmbH, Martinsried, Germany; 3 Defence Research and Development Canada Suffield Centre, Department of National Defence, Suffield, Canada; Institut Pasteur, FRANCE

## Abstract

**Background:**

Molecular diagnostics has become essential in the identification of many infectious and neglected diseases, and the detection of nucleic acids often serves as the gold standard technique for most infectious agents. However, established techniques like polymerase chain reaction (PCR) are time-consuming laboratory-bound techniques while rapid tests such as Lateral Flow Immunochromatographic tests often lack the required sensitivity and/or specificity.

**Methods/Principle findings:**

Here we present an affordable, highly mobile alternative method for the rapid identification of infectious agents using pulse-controlled amplification (PCA). PCA is a next generation nucleic acid amplification technology that uses rapid energy pulses to heat microcyclers (micro-scale metal heating elements embedded directly in the amplification reaction) for a few microseconds, thus only heating a small fraction of the reaction volume. The heated microcyclers cool off nearly instantaneously, resulting in ultra-fast heating and cooling cycles during which classic amplification of a target sequence takes place. This reduces the overall amplification time by a factor of up to 10, enabling a sample-to-result workflow in just 15 minutes, while running on a small and portable prototype device. In this proof of principle study, we designed a PCA-assay for the detection of *Yersinia pestis* to demonstrate the efficacy of this technology. The observed detection limits were 434 copies per reaction (purified DNA) and 35 cells per reaction (crude sample) respectively of *Yersinia pestis*.

**Conclusions/Significance:**

PCA offers fast and decentralized molecular diagnostics and is applicable whenever rapid, on-site detection of infectious agents is needed, even under resource limited conditions. It combines the sensitivity and specificity of PCR with the rapidness and simplicity of hitherto existing rapid tests.

## Author summary

Rapid and reliable on-site diagnostics are an essential part of infectious disease outbreak response and the fight against neglected tropical diseases. In this respect, molecular diagnostics represents the current gold standard. However, the vast majority of current molecular diagnostic methods (such as Polymerase Chain Reaction) are lab-bound techniques, require expensive and heavy instrumentation and are time consuming to perform. Moreover, in resource limited countries with little or no infrastructure, samples often have to be transported over long distances to the few reference laboratories capable of such diagnostics. This significantly slows down the entire diagnostic process, thus considerably delaying important decisions necessary to contain outbreaks and to initiate medical countermeasures. Until now, there are only few rapid on-site diagnostic tests for a limited number of infectious agents available and they often lack sensitivity and/or specificity. Here we present a new technology–pulse-controlled amplification–which enables rapid (<20minutes) and portable, yet sensitive and specific on-site molecular diagnostics based on nucleic acid amplification. The device we developed is lightweight, battery operated and we demonstrate that our tests can be performed under resource limited conditions and without nucleic acid extraction, even when wearing full personal protective equipment. Thus, molecular diagnostics can be carried out on-site in a non-laboratory environment without the need for nucleic acid extraction. We envision that this new technology has the potential to become a gold standard method in nucleic acid detection for front-line and in-field applications.

## Introduction

Infectious diseases caused by pathogenic microorganisms such as bacteria, viruses, fungi or parasites, are still among the top ten causes of death worldwide [1]. Rapid and specific diagnostics are an essential pillar of every outbreak-response and have the power to prevent epidemics or even pandemics. One infamous example is pneumonic plague caused by *Yersinia pestis*, a category A bioterrorism agent [2], which can be fatal if not treated within 24 hours after the onset of symptoms. As of today, about 75% off all global plague cases occur in Madagascar. In 2017, Madagascar experienced an unusual wide-spread plague epidemic that also affected large cities [3]. During this outbreak immunochromatographic rapid tests were used for decentralized diagnostics. However a recently published study revealed that the tests used lack both sensitivity and specificity [4]. In addition, the only laboratory able to provide confirmatory diagnostics is located in the capital of Madagascar, Antananarivo. Due to the often prolonged sample transport times from plague foci to the laboratory combined with the time consuming cultivation-based reference test, it can take several days to get the final results [5]. For plague outbreaks in China it has even been reported that it can take up to 15 days to obtain final laboratory results [6], further illustrating the urgent need for reliable decentralized diagnostics. This is also confirmed by Rajerison et al., stating that “improving all tools for plague diagnosis, including those suitable for point-of-care screening in remote areas remains a research priority to control human plague” [4]. Besides the risk of natural outbreaks, modern threats of bioterrorism have the potential to cause mass casualty incidents of serious infectious diseases, and therefore also require quick and reliable diagnostic tests that can be performed on-site rather than in a centralized laboratory [7]. Thus, in modern diagnostics of tropical- and epidemic-prone infectious diseases, rapid diagnostic tests (RDTs) are indispensable assets, but have to live up to high expectations: they need to be portable and easy-to-use, while at the same time deliver results with high sensitivity and specificity in little time to facilitate a reliable diagnosis for adequate therapy.

Lateral flow assays (LFAs) are a common example of RDTs and come in several different formats. Principally, they all use immunochromatography to detect the presence or absence of a specific target analyte in a liquid sample without the need for intensive sample preparation and additional equipment. They are small and portable devices that deliver rapid (i.e. 15–20 minutes) results, thereby moving diagnostic testing away from centralized laboratories and closer to the patient or even directly into the field. Thus, they have become an indispensable technique in point of care (POC) testing and are used by first responders worldwide [8,9]. However, data on sensitivity and specificity of LFAs is limited but generally points to high detection limits and often lack of specificity [4,10,11]. Furthermore, sensitivity of detection methods based on antigen recognition is dependent on the permanent presence and sufficient concentration of the specific antigen. LFAs for the detection of *Y*. *pestis*, for example, use the F1 capsular protein. However, the F1 gene is temperature regulated and only expressed at ≥33°C, which can lead to false negative results if the tests are used in an environmental or bioterrorist context [12].

In contrast to LFAs, polymerase chain reaction (PCR) is a highly sensitive method with great specificity and reproducibility and many gold-standard protocols have been established for a broad range of biological agents. During the last decade, a variety of PCR-based techniques have been developed to further improve diagnostic capabilities, including real-time quantitative PCR (qPCR), multiplex qPCR and digital droplet PCR (ddPCR) [13]. Nevertheless, real-time PCR remains a time-consuming and laboratory bound technique, usually requiring DNA extraction prior to amplification and often large, heavy, and power-consuming thermal cyclers. Cartridge based systems such as the GeneXpert or the BioFire FilmArray overcome some of these restraints but still rely on landline power supply, lack the possibility to process several samples at once and are highly expensive (e.g. the BioFire BioThreat-panel, the only panel for this platform that includes *Y*. *pestis*, costs 150€ per reaction). Several other nucleic acid amplification methods are available which have the potential to become point of care detection and diagnostics tools, mostly relying on isothermal amplification methods such as Loop-mediated isothermal amplification (LAMP). However, they often require multiple primers thus highly aggravating the detection of pathogens with highly divergent sequences, such as Filoviridae [14]. Furthermore, due to the required large number of individual primers these assays are particularly prone to primer-dimer formation, which in some cases even lead to false positive results [15].

To address these different limitations that existing methods present, we introduce an ultra-fast, yet sensitive and specific method for the nucleic-acid based detection of infectious agents, i.e. pulse- controlled amplification (PCA). As a proof of concept we developed a PCA-assay for the detection of *Y*. *pestis* under standard lab-conditions using both purified DNA and crude culture material as samples. Furthermore, we illustrate its applicability as a POC test for clinical samples without prior nucleic acid extraction (using *Y*.*pestis* spiked sputum samples). Finally, we also demonstrate the field usability of PCA in a bioterrorism context wearing heavy personal protective equipment, thus illustrating a broad range of possible applications of PCA relevant for the diagnostics of neglected tropical diseases such as pneumonic plague.

## Material and methods

### Pulse-controlled amplification

**Fig 1:**
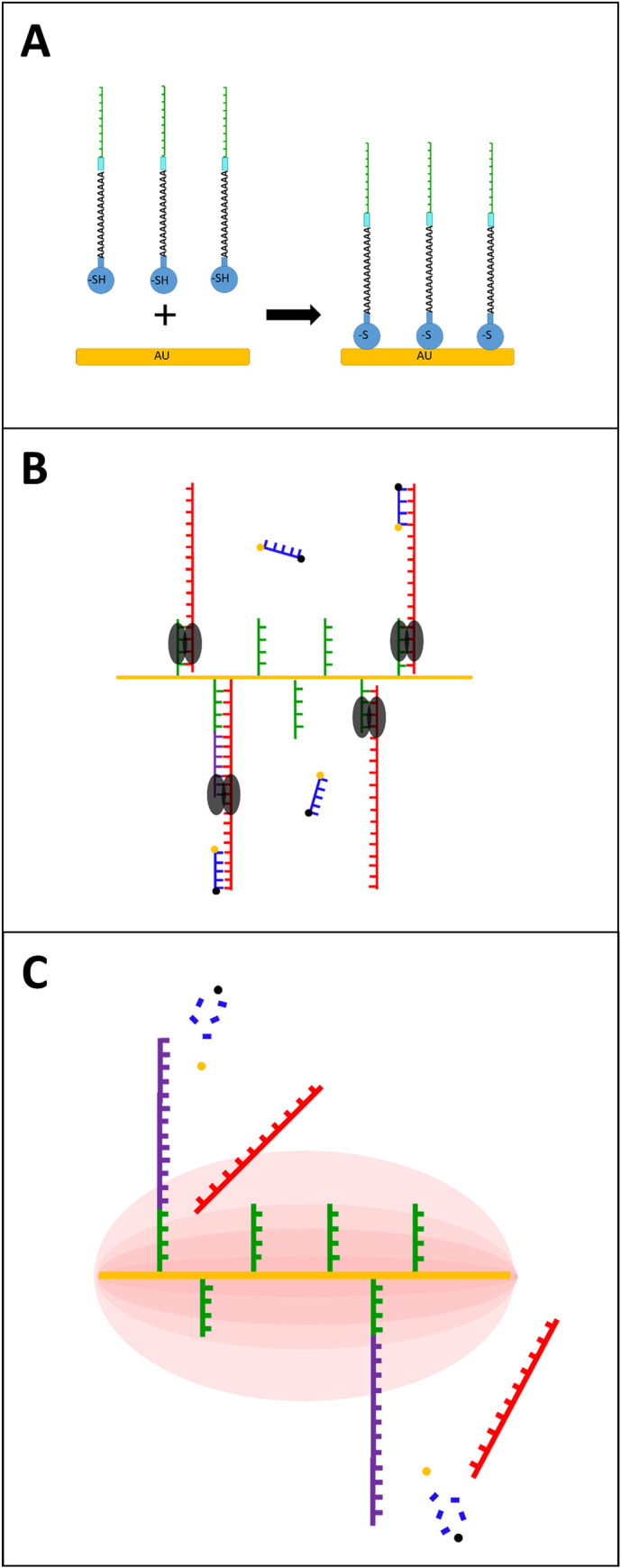
**Schematic of the PCA process and the interaction of primers and gold coated wires (A)** (reduced) **Thiol-modification** was added to the 5’-end of the **primer** using a **poly-A-tail** and **Int Spacer 9**, allowing for a strong AU-S bond and immobilization of the primer to the gold-coated wires **(B)** Annealing, **probe** hybridization and Elongation **(C)** PCA uses thermal cycling but instead of time-consuming alternating heating and cooling of the whole reaction mixture, rapid energy pulses are applied to the gold wires, causing ultra-fast local heating and dsDNA denaturation.

Similar to PCR, pulse-controlled amplification (PCA) relies on the exponential amplification of a specific nucleic acid target fragment for subsequent detection. Amplification is achieved through the binding of target-specific, complementary oligonucleotide primers to template DNA, followed by primer extension by a DNA polymerase enzyme. PCA also relies on thermal cycling, however, instead of time-consuming alternating heating and cooling of the total reaction volume (“global heating”), rapid sub-millisecond voltage pulses are applied to an array of 75 gold-coated tungsten wires (15 μm diameter, 200 nm Au coating), causing ultra-fast heating within only a micrometer-sized liquid layer surrounding each wire (“local heating”). The remaining bulk of the reaction volume (more than 99%) is kept at the base temperature used for annealing and elongation. The approach of “local heating” denatures double stranded (ds) DNA only within the heated layer surrounding the wires, which makes it necessary for part of the reaction to be localized as well. This is achieved by attaching one of the primers to the micro-scale conductive metal structures (in this study gold-coated tungsten wires). The other primer remains free in solution, providing the kinetic advantages of a free reaction. As a result, the dsDNA denaturation step of the amplification reaction requires only a fraction of the energy usually required to thermocycle the total reaction volume. Local heating allows the wires to cool off after the voltage pulse that drives the denaturation step by thermal diffusion on a millisecond time scale. The bulk of the reaction volume serves as cooling reservoir for an entirely passive cooling process of the embedded wires, resulting in ultra-fast thermal cycles. This reduces the total time of the amplification process by a factor of up to ten compared to PCR, as hundreds of energy pulses can take place in a short amount of time. Like qPCR, amplification can be traced in real time using intercalating dyes or, as in our study, hydrolysis probes (Fig 1).

Currently, PCA is performed on a prototype instrument, the Pharos Micro (GNA Biosolutions, Martinsried, Germany) utilizing prototype disposable chips, which contain the amplification reactions (GNA Biosolutions, Martinsried, Germany). To optimize assays, different parameters of the run are adjustable, including base and lid temperature of the Pharos Micro [°C], heating time [μs], cycle time [s], number of cycles, and thermalizing time [s]. For primer design, the guidelines in Table 1 should be followed. For successful PCA, it is critical to avoid primer dimers when designing primers, especially for the thiolated-primer used for functionalization of the wires.

**Table 1:** Guidelines for PCA primer and probe design.

GC content primer and probe	40–45% (max 60%)
Primer length	20–30 bases
Probe length	20–30 bases
Amplicon length	Optimum 70–120 bp (max 350 bp)
Melting temperature primer (6mM MgCl2, 30 mM NaCl, 500 nM primer)	61–65°C

### Pharos micro prototype and chip design

**Fig 2:**
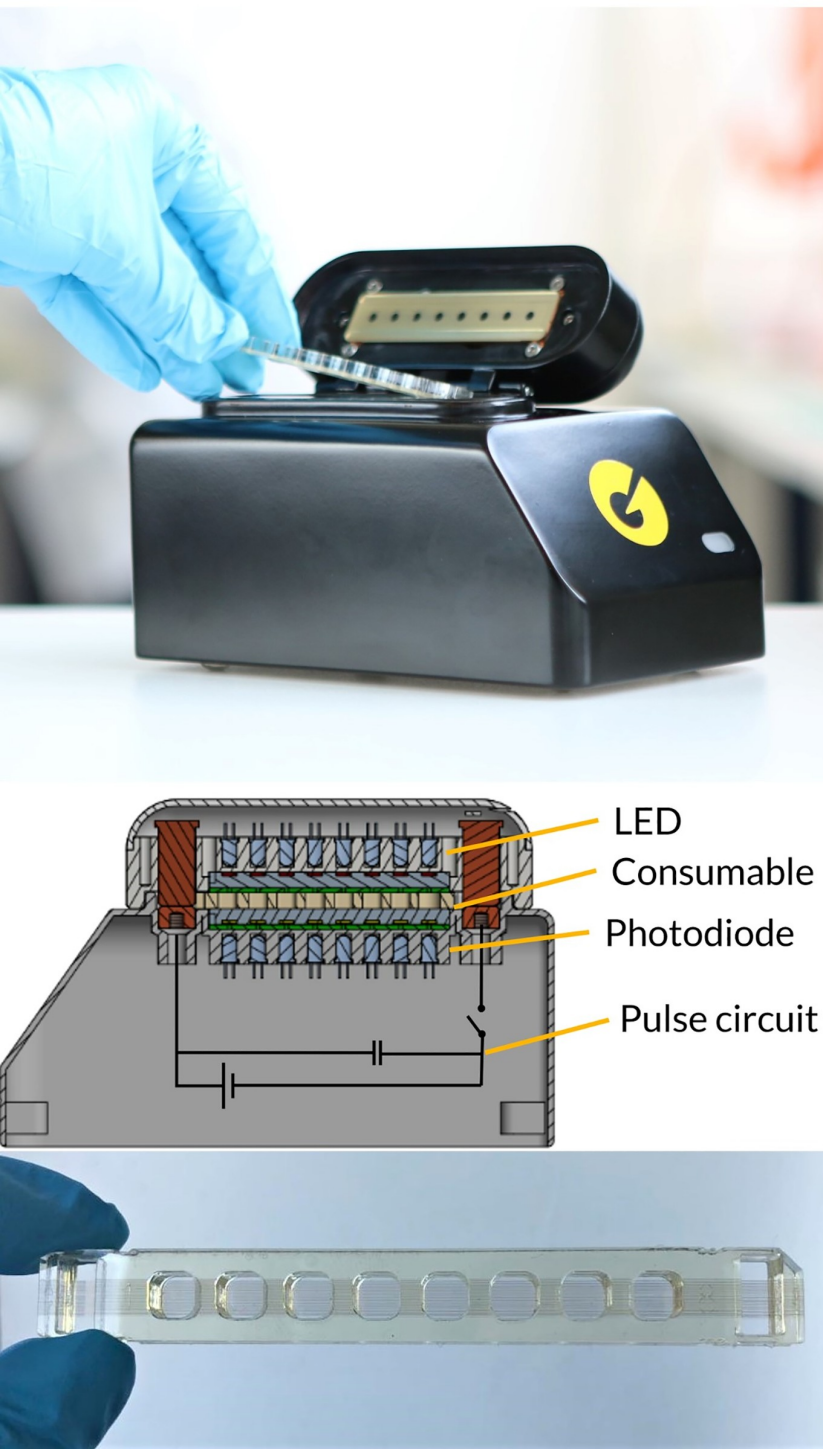
Pharos Micro prototype. **Upper panel:** The current model 8-well duplex manual sample-to-answer prototype system for research and assay development, using the PCA approach. **Middle panel:** Schematic view (right) showing the concept of the instrument: The disposable chip (yellow) is sandwiched between two heat blocks (grey) used to set the base temperature. Transmission fluorescence measurements (multicolour LEDs on top, photodiodes on the bottom) for hydrolysis probe chemistry as well as a schematic of the circuit to drive PCA, which basically only requires a capacitor and a fast switch (i.e. a MOSFET) to deliver the pulses necessary for localized heating. **Lower panel:** prototype chip. Each PMMA-chip has eight wells with 75 ultra-thin gold-coated tungsten micro wires running through the entire chip at the bottom of every well.

The Pharos Micro prototype (GNA Biosolutions, Martinsried, Germany) used in this study consists of a 3D-printed housing and lid with a size of 100mm x 175 mm x 110 mm (WxDxH) and weighs 900 g (Fig 2). The instrument is equipped with light-emitting diodes and filters for real-time fluorescence detection and electronic control modules. Two conventional heating blocks are set to a constant temperature at the bottom and the top of the chip to maintain the reaction volume at a constant temperature of 65°C for annealing and elongation. The Pharos Micro prototype is battery-powered for field use (using a commercially available power bank) or can be connected to a power supply (230 V) in the stationary lab. It is operated with a dedicated software (GNA Biosolutions, Martinsried, Germany) on a tablet (or laptop) connected to the instrument via USB. The prototype disposable test chips consist of Poly(methyl methacrylate) (PMMA) and have eight wells, with each one fitting 40–80 μl reaction volume (Fig 2). All wells are equipped with 75 paralleled, gold-coated tungsten micro wires of 15 μm diameter resulting in a resistance of ≈500 *m*Ω. The wires allow for the electrical contact to the Pharos Micro prototype at both ends. Primers are coupled to the wires via a functionalization step. Chips are sealed with an adhesive tape after sample loading. More details on the prototype can be found under supporting information (S1 Text).

### Functionalization of chips

Chips were functionalized with the (thiol-modified) forward primer. Primer was diluted in functionalization buffer 2 (GNA Biosolutions, Martinsried, Germany) to a final concentration of 500 nM. 50 μl of primer dilution was loaded into each well and incubated for 20 minutes at room temperature. Functionalization solution was removed and wells were washed five times with demineralized water. After washing, chips were tapped out and the dry chips were stored at 4°C until further use. Chips were prepared fresh every morning, however they can be stored for up to five days without significant loss of efficiency. The Chips are single use only.

### *Yersinia pestis* pla PCA assay

*Y*. *pestis* pla gene specific primers and probes were designed based on alignment studies using BLAST and the sequence database of the National Centre for Biotechnology Information (NCBI). The primer pair and probe showing best results in pilot experiments were used for PCA experiments (Table 2) Primers and probe were obtained from Ella Biotech (Martinsried, Germany). The forward primer was modified as depicted in Table 2, including a Thiol-modification, a poly-A portion and an internal spacer. All reactions were performed using a freeze-dried mastermix (GNA Biosolutions, Martinsried, Germany), containing all necessary components. Optimal concentrations for all components were determined in pilot experiments and final concentrations are shown in Table 3. For PCA, mastermix was dissolved in the appropriate amount of DNAse-free water to achieve a final reaction volume of 40 μl.

**Table 2:** PCA-primers and probes used in this study.

Name	Target	Sequence 5’– 3’	Size (bp) of PCA product
**YPpla_F**	*Y*. *pestis* pla gene	Thiol-A_35_/iSp9/ACGGCGGGTCTGCAATA	81
**YPpla_R**		ACAGACATCCTCCCCGCT	
**YPpla_TM**		FAM-CGCTTCTGAAAAATACAGATCATATCTCTCTTT-BHQ1	

**Table 3:** Optimized composition of the freeze-dried mastermix.

Component	Final concentration
**MgCl**_**2**_	3 mM
**5x Mastermix** *	1x
**5x Boostermix** *	1x
**YPpla_R**	500 nM
**YPpla_TM**	50 nM

* GNA Biosolutions (Martinsried, Germany)

For a PCA run the following parameters were used: 10 s thermalization, 550 cycles, 1.5 s cycle time, 73°C lid temperature, 66°C base temperature, 350 μs heating.

For samples containing purified genomic DNA as template, lyophilized mastermix was dissolved in 316.8 μl DNAse-free water. 36 μl of mastermix was loaded into each well of the chip and 4 μl of template was added. When using bacterial culture material and spiked sputum as samples, an initial thermal lysis and binding of the target DNA to the primers attached to the wires was performed prior to PCA: 60 μl/well of liquid culture material was loaded. Chips were sealed and placed in the Pharos Micro for 5 min (with 66°C base temperature). After incubation, culture material was removed and discarded, lyophilized mastermix was dissolved in 352 μl DNAse free water and 40 μl was added directly to each well.

### Determination of precision and limit of detection

pla copy numbers of serial dilutions of purified DNA as well as crude culture spiked sputum material were determined by ddPCR. The sensitivity and reproducibility of PCA were then investigated for all three sample types and the 95% limit of detection (LOD) was determined with standard probit regression analysis (S1 Fig) using the STATGRAPHICS Centurion 18 software (Version 18.1.12) and following the MIQE guidelines [16]. Each PCA was performed three times with six replicates for each tested dilution and sample type.

### Lateral flow assay

We used the miPROTECT Plague (Miprolab, Göttingen, Germany) lateral flow assay as the standard reference method for the detection of *Y*. *pestis* in liquid culture under field conditions. Tests were performed according to the protocol provided by the manufacturer and results were obtained after 20 minutes.

### Bacterial culture

*Y*. *pestis* strain EV76 was used as the model organism in this study. Liquid cultures were grown in LB–bouillon at 37°C. For comparability of experiments, *Y*. *pestis* cultures were diluted to a McFarland Standard 0.5 followed by serial 10-fold dilutions in LB-medium which were then used for experiments. Dilutions were prepared fresh every day.

For experiments with inactivated sample in the field exercise, a fixation of culture material was performed with 4% paraformaldehyde (PFA). For that, 1 ml of liquid culture was centrifuged at 14,000 rpm for 2 minutes. The supernatant was discarded and the cell pellet was resuspended in 1 ml PBS and centrifuged again. The supernatant was discarded, the cell pellet was resuspended in 250 μl PBS and 750 μl 4% PFA and fixated at 4°C overnight. To remove PFA from the fixated sample, it was centrifuged at 14,000 rpm for 2 minutes, supernatant was discarded and the cell pellet was resuspended in 1 ml PBS and stored at 4°C until further use. In order to simulate a contaminated culture in the clandestine laboratory and to challenge the assay with high non-target background the target cells were mixed in a 1:100 ratio with *Escherichia coli*.

For experiments with simulated patient material, sputum samples (anonymized diagnostic left-overs) were spiked with *Y*. *pestis* strain EV76 overnight cultures. Cultures were diluted to a McFarland Standard 0.5 (1ml), centrifuged (14,000 rpm, 2 minutes) and pellets were resuspended in sputum (1ml) followed by 10-fold dilutions in sputum material.

### DNA preparation

For DNA extraction, 1 ml of a liquid culture of *Y*. *pestis* with McFarland 0.5 was used. Purified DNA was prepared using the QIAamp DNA Mini Kit (Qiagen, Hilden, Germany) according to the manufacturer’s instructions. DNA was eluted in 50 μl of elution buffer and stored at -20°C until further use. DNA concentration was determined using the Qubit dsDNA high-sensitivity (HS) quantification assay according to the manufacturer’s instructions. Measurement was performed using a Qubit 3.0 fluorometer (Invitrogen, Carlsbad, USA).

### Real-time quantitative PCR (qPCR)

qPCR was used as a reference method for the amplification of *Y*. *pestis* DNA. qPCR was performed as previously described by Riehm et. al. using the pla target region [17]. In this study, sample volume was 5 μl and the assay was performed using a Rotor-Gene Q 2plex (Qiagen, Hilden, Germany).

### MinIon 16S sequencing

For 16S Sequencing of purified sample DNA, the library was prepared using the Oxford Nanopore16S Barcoding Kit (SQK-RAB204) according to the manufacturer’s protocol (Oxford Nanopore Technologies, Oxford, UK). Sequencing was performed on a MinIon using a flow cell and base calling was performed using a MinIT (Oxford Nanopore Technologies, Oxford, UK).

### Digital droplet PCR

ddPCR was used to accurately quantify copy numbers (pla) and target cells in the samples used in this study. As our PCA assay uses the pla-gene on the multicopy pPCP1 plasmid as the target sequence for the detection of *Y*. *pestis*, digital droplet PCR (ddPCR) was used to accurately determine the pPCP1 plasmid copy number in the EV76 strain used in this study: *Y*. *pestis* genome copy numbers were determined using an EvaGreen based ddPCR. In contrast, for the quantification of the pPCP1 plasmid a pla-specific ddPCA was performed and copy numbers were compared to determine number of plasmids per bacterial cell. All quantifications were performed in triplicates and on three biological replicates.

Primers and probes were used as described by Riehm et. al.[17] for pla-specific ddPCR and were obtained from TIB MOLBIOL (Berlin, Germany). The 20 μl ddPCR reaction mixture per sample contained 10 μl 2X Supermix for Probes (no dUTP), 1.8 μl of each primer (10 μM μl^-1^), 0.5 μl probe (10 μM μl^-1^) and 2 μl template. EvaGreen ddPCR was performed as previously described by Ziegler et. al. [11].

Droplets were generated using the BioRad QX100 Droplet Generator Hercules, USA) and PCR was performed on the Veriti 96-well Thermal cycler (Applied Biosystems, Foster City, USA) using the following reaction conditions:

pla: 10 min at 95°C, followed by 40 cycles of 30 s at 95°C and 1 min at 60°C and a slope of 2°C s^-1^. For enzyme deactivation, a final heating step of 10 min at 98°C was performed at the end.

EvaGreen: 5 min at 95°C, 40 cycles of 30 s at 95°C and 1 min at 60°C and a slope of 2°C s^-1^. For signal stabilization and enzyme inactivation, a final cooling and heating step was performed at the end with 5 min at 4°C and 5 min at 90°C.

ddPCR was evaluated using the BioRad QX100 Droplet Reader (Hercules, USA) and the corresponding QuantaSoft Analysis Pro software.

## Results

### pPCP1 plasmid copy numbers

For the *Y*. *pestis* strain EV76 used in this study the pPCP1 plasmid copy numbers per target cell were determined by ddPCR. Target cell quantification by EvaGreen ddPCR revealed 6.5 x 10^7^ cells per ml (McFarland 0.5). pla specific ddPCR of the same samples revealed an average of 3.4 x 10^9^ pPCP1 plasmids per ml, resulting in an average of 52.3 pPCP1 plasmids per target cell.

### Detection of *Y. pestis* under standard laboratory conditions

**Fig 3:**
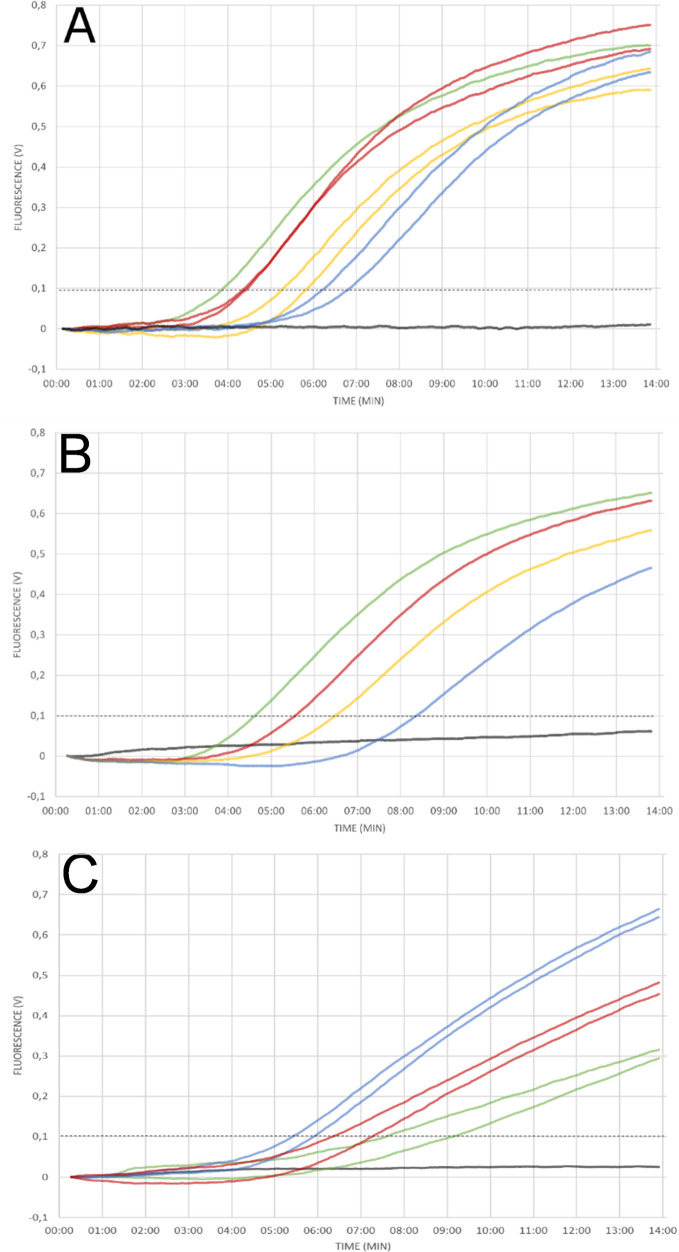
Detection of *Y*. *pestis* pla gene. **(A)** Different DNA concentrations were tested in duplicate reactions using purified DNA as template. Exponential amplification was observed for the positive control containing 10^8^ copies (green), and all template containing samples: 10^7^ (red), 10^6^ (orange), 10^5^ (blue) copies per reaction. No amplification was observed for the negative control (black). The onset of amplification did not clearly depend on the logarithm of the copy-numbers used, indicating a semi-quantitative quality of PCA **(B)** *Y*. *pestis* specific PCA was performed with crude sample material and an initial 5-minute hybridisation step prior to PCA. Exponential amplification was observed for positive control (green), undiluted sample (3,9 x 10^6^ cells per reaction; red), 1:10 dilution (yellow) and 1:100 dilution (blue) of sample. No amplification was observed for sample containing no bacterial culture material (black) **(C)** PCA was performed in duplicates with different dilutions of *Y*. *pestis* in sputum samples (crude sample material). Amplification was observed for all dilutions (1:10 blue, 1:100 red) of spiked sputum samples (red&yellow) down to 8 x 10^2^ cells per ml (≈48 cells per reaction).

We applied PCA to *Y*. *pestis*, using the pla gene as target and purified genomic DNA as template. While the negative control did not produce a signal, amplification was observed in all template-containing samples (in duplicates). The detection threshold was reached in 03:54 minutes (10e8 copies) and 6:50 minutes (10e5 copies) and the entire PCA run was completed in 13.75 minutes. However, the onset of amplification did not clearly depend linearly on the logarithm of the initial copy-numbers indicating that PCA in its current implementation is a qualitative or only semi-quantitative method (Fig 3A).

To assess PCA’s field and POC applicability, i.e. testing of samples without prior nucleic acid extraction, we applied the same PCA protocol to 10-fold serial dilutions of crude samples (culture material, spiked sputum) of *Y*. *pestis* but added an additional 5-minute incubation step at 66°C base temperature. In preparation of the field testing during which only inactivated sample could be used, initial experiments were performed with PFA-fixated culture material and clean amplification was observed for all tested log-dilutions of sample while negative controls did not produce any signal. Subsequently, all experiments were repeated with 10-fold serial dilutions of non-fixated culture material and sputum samples spiked with *Y*. *pestis* following the same protocol. Again, amplification could be observed for all target cells containing samples whereas no amplification was observed for negative controls (Fig 3B and 3C).

### Analytical precision and limit of detection

Results of the probit regression analyses revealed a LOD (95%) of 434 copies per reaction for purified DNA and 35 cells per reaction (≈583 cells/mL) for crude culture material and indicated efficient reproducibility of the assay. Detection of lower copy numbers was possible but less reproducible for the pla PCA assay.

### Evaluation of specificity

To determine specificity of the pla assay, PCA was performed in duplicates with purified DNA of various bacteria listed in S1 Table. Based on whole genome sequences, *Y*. *enterocolitica* and *Y*. *pseudotuberculosis* are classified as nearest phylogenetic neighbors [18]. For these closely related species, specificity was tested in triplicates with not only purified DNA but also bacterial culture material. For all bacteria tested, PCA was negative, confirming the specificity of the assay.

### Evaluation of field handling

**Fig 4:**
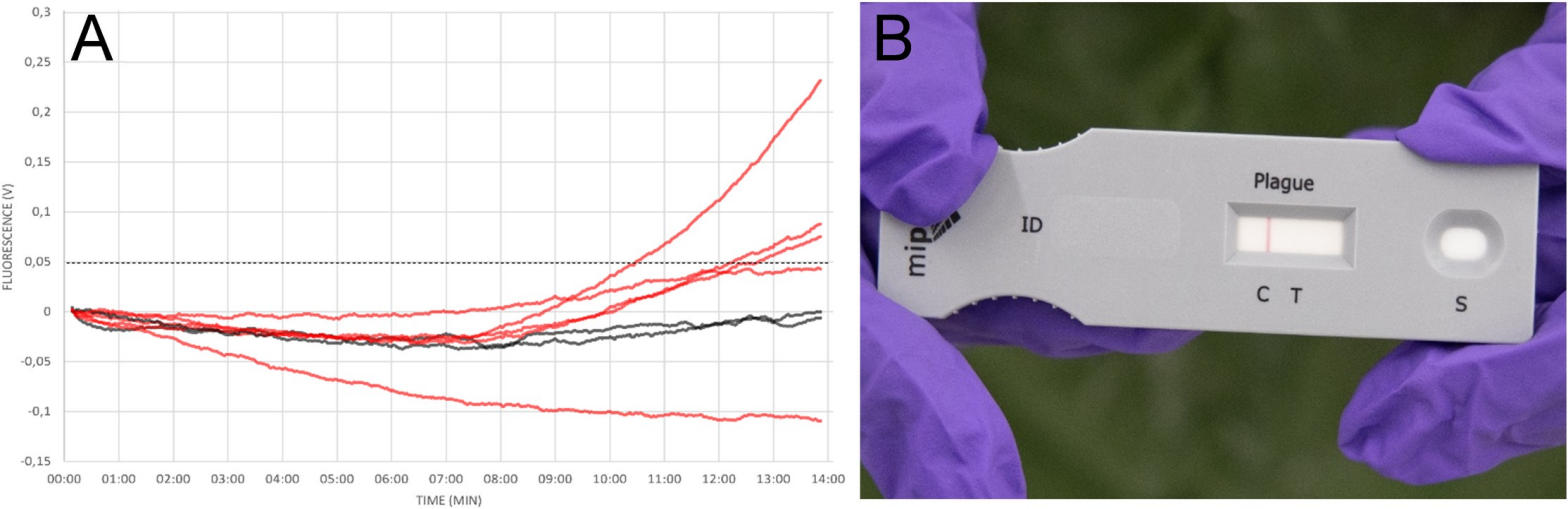
PCA can be performed under field conditions. PCA and LFA specific for *Y*. *pestis* were performed in parallel under field condition wearing heavy Personal Protective Equipment (PPE). Results were obtained within 20 minutes for both tests. **(A)** PCA: negative controls (black), sample (red) **(B)** LFA: test was valid as indicated by the control line but was negative for *Y*. *pestis*.

To evaluate feasibility of the method under field conditions (i.e. testing directly at sampling site, battery operated, no prior nucleic acid extraction, operators wear PPE), both PCA and LFA were performed in parallel in a training scenario during the international CBRN-live agent exercise Precise Response 2019 in Suffield, Canada. In this scenario a clandestine laboratory was searched by special forces wearing fully encapsulated PPE. A turbid liquid broth incubated at 28C° and scientific literature evidence pointed towards an attempt to grow *Y*. *pestis*. After sampling of the liquid bacterial culture, both tests were performed simultaneously. Results were obtained after 20 minutes. While the LFA was unable to detect *Y*. *pestis* (Fig 4B)—indicating that there are less than 10^4^ target cells per ml in the analyzed sample [11]—PCA was positive in three out of five replicates, while both negative controls did not produce a signal (Fig 4A). The sample was then analyzed in our stationary laboratory. After DNA extraction, qPCR and 16S sequencing was performed and confirmed presence of *Y*. *pestis* in the acquired sample material along with large amounts of *Escherichia coli* (S2 Fig).

## Discussion

PCA is a novel method based on the principle of PCR, which can effectively be used as RDT in the detection of infectious agents in resource limited environments. To compare PCA and LFA directly, an assay for the detection of *Y*. *pestis* was developed. Since PCA is based on nucleic acid amplification and detection, *Y*. *pestis* pla gene was used as target. Because of its high copy number among the Orientalis biovar (52 copies for the EV76 strain used in this study), *pla* is a commonly used target [17,19]. However, it is important to consider that as pla may be found in other *Enterobacteriaceae* such as *Citrobacter koseri* and *Escherichia coli* [20], a confirmatory PCR with additional targets (e.g. caf1 [21]) should be applied. Additionally, new specific assays targeting multi-copy targets such as 16S or 23S ribosomal RNA should be designed for PCA.

The results in this study show that PCA can be applied to both purified and crude sample material. The fast and easy workflow (approximately 20 minutes from start to finish) is comparable to the time required for most LFAs. Furthermore, we demonstrated the applicability of this quick workflow for crude sample material, including sputum samples–a highly relevant clinical sample matrix for pulmonary plague diagnostics [4]. For the field handling test we additionally challenged the prototype device by mixing the target bacteria with a 1:100 ratio of non-target bacteria (*E*. *coli*), thereby simulating a contaminated culture in the clandestine laboratory. PCA was capable of detecting the target organism even within this massive non-target background. Even though we operated the device in the field under challenging conditions and at the limit of detection (<10^4^ target cells per ml mixed 1:100 with non-target cells), the fact that 3 out of 5 replicates were positive, while the simultaneously conducted LFA was negative, illustrates the advantages over LFA of this technology and its potential for field applications. In the current set-up–especially with pla as the only target for *Y*. *pestis* detection—such a result would of course need confirmation, for instance by real-time PCR or by implementing a multiplex assay with additional targets. However, field results are always regarded as presumptive results and mandatorily require confirmation.

The LOD (95%) of purified DNA was 434 copies per reaction, however since LFAs are antigen-based tests, results cannot be compared directly. As an indirect comparison, we determined that PCA is more sensitive in detecting *Y*. *pestis* in culture material with LOD (95%) of 583 cells/mL, which is a result of the high gene dose effect. This LOD is well below the described LOD for *Y*. *pestis* LFA–as illustrated by the negative LFA, which we simultaneously conducted in the field. When compared to pla-specific qPCR on the other hand, it becomes clear, that qPCR is more sensitive with published LODs (95%) of as low as 0.1 genome equivalents [22]. However, as for PCR, sensitivity of PCA is assay dependent. Another recently published study developed a PCA assay for the detection of SARS-CoV-2, illustrating the possibility to develop reverse transcriptase PCA assay to detect RNA-Viruses [23]. When comparing time to result, PCA is much faster in obtaining results (13.75 min total run time) than Real-time PCR (> 60 minutes). As demonstrated in the field exercise the time from taking the sample to result is only 20 minutes and thus matches the time required for the simultaneously conducted LFA. Compared to real-time PCR the results are only semi-quantitative, but still provide more quantitative information than LFAs. Taken together, the above results demonstrate that as a molecular diagnostic technology, PCA combines some of the most advantageous elements of both LFA and PCR: (I) it is more sensitive and specific than LFAs (II) it is as specific as PCR, (III) it takes a fraction of the time needed for PCR and (IV) in contrast to PCR it can be performed on an affordable, battery powered, lightweight and thus portable platform that enables testing in extra-laboratory or non-traditional laboratory environments as well as Point of Care. As previously described [6] freeze-drying of PCR chemistry is an essential factor for stability and field use. Accordingly, all critical components of the plague assay used in this study were freeze-dried.

Due to its speed and portability, PCA facilitates sensitive nucleic acid-based detection of infectious agents in a non-laboratory POC environment. In 2003, the WHO identified seven ideal characteristics of tests that all newly developed POC diagnostics should meet. Based on the acronym ASSURED, these tests should be **A**ffordable, **S**ensitive, **S**pecific, **U**ser-friendly, **R**apid & robust, **E**quipment-free and **D**elivered [24]. However, even in 2020, hardly any tests exist that meet these criteria. While LFAs meet some of the criteria outlined by WHO, they greatly lack sensitivity and specificity as previously described. PCR on the other hand is neither rapid, nor equipment-free. In addition, it requires expensive instrumentation and trained medical staff to be performed and evaluated. Moreover, high power consumption prevails battery operation of all real-time PCR devices, whereas the PCA platform only requires 8W per run and thus can be powered by solar chargeable power banks. The PCA platform will cost less than 15 TSD € and thus will be cheaper than the FilmArray or most real-time PCR machines. One reaction costs approximately 7€ and thus is considerable cheaper than one lateral flow assay which typically cost between 35 to 49€ per test. PCA is rapid, sensitive, specific, and it requires only minimal equipment and is relatively simple to perform. The field protocol for example relies on single use Pasteur pipettes and requires only a minimum of pipetting steps. However, PCA does require initial training to perform and evaluate an assay.

Due to the simplicity of PCA assay design, which requires the same number of primers as conventional PCR, it may be possible to transfer the majority of existing real-time PCR assays to the PCA format with little or no modifications/optimizations. Moreover, in conventional PCR, nucleic acid extraction methods are often necessary prior to amplification and detection–thereby prolonging the whole procedure and limiting its field applicability. PCA technology is also suitable for the multiplex format (currently two channels: FAM and Cy5). While other commercially available platforms, such as the FilmArray [25] allow for highly efficient multiplex screening for several pathogens, their power requirements render them lab-bound, thus they are not true point-of-care or point-of-incidence technologies. In addition, the high instrument- and assay costs further prevent widespread use of such devices, especially to third world countries. Due to the fact that PCA, in contrast to many “closed” cartridge systems such as FilmArray or GeneXpert, is an open-source system, existing in-house real-time PCR assays can easily be transferred to PCA for diagnostics. Of course, not all sample matrices are equally suitable for direct PCA without prior nucleic acid extraction. Whole blood for example as well as untreated soil-samples did not work well in direct PCA. However, there are rapid and instrument free nucleic acid extraction protocols available which presumably facilitate POC analyses of such challenging matrices [26].

In conclusion, we demonstrated a novel ultra-fast method for the amplification and detection of nucleic acid through Pulse Controlled Amplification (PCA). PCA combines the speed of LFAs or isothermal methods, with the simplicity of assay design of PCR, in a novel portable and battery operated system. This work successfully demonstrates example applications for PCA in the detection of *Y*. *pestis*, under laboratory, point-of-care and field conditions. It expands the use of molecular testing to extra-laboratory or non-traditional laboratory settings, as well as near-patient setting, and has the potential to become a powerful technology in nucleic acid detection for front-line and in-field applications.

## Supporting information

S1 Text Technical information on the Pharos micro prototype. (DOCX)

S1 Fig Analytical limit of detection. **(A)** Probit regression analysis (left) reveals a LOD (95%) of 434 pla copies per reaction for purified DNA. PCA results (right) of samples containing 1x10^**4**^ (red) copies per reaction and 413 (yellow) copies per reaction (negative control, black). **(B)** Probit regression analysis (left) reveals a LOD (95%) of 1824 pla copies per reaction. Considering that the EV76 strain used in this study was shown to contain 52 pPCP1 copies, this equals 35 cells per reaction. PCA results (right) of crude culture material containing 3.9x10^**4**^ (green) copies per reaction (≈750 cells) and 1800 (yellow) copies per reaction (≈34.6 cells).
(TIF)

S2 Fig Conventional qPCR and 16S sequencing confirm *Y*. *pestis* in field sample (A) Sample DNA was extracted and subjected to conventional pla specific qPCR in our stationary laboratory. Presence of *Y*. *pestis* was conformed (sample: yellow, positive control: red, negative control: blue) (B) Krona chart: Subsequent 16S sequencing of the sample revealed large amounts of contaminating bacteria (>95% *Enterobacteriacea*) and only little amounts (0.5%) of *Y*. *pestis*.
(TIF)

S1 Table No cross-reactivity was observed with any of the tested bacteria, confirming specificity of the pla assay (ATCC: American Type Culture Collection; DSMZ German Collection of Microorganisms and Cultures; NCTC: National Collection of Type cultures). (XLSX)
