# Supplementary material for: Pulse-Controlled Amplification–A new powerful tool for on-site diagnostics under resource limited conditions
Source: PLoS Negl Trop Dis. 2021 Jan 29;15(1):e0009114. doi: 10.1371/journal.pntd.0009114 (PMC7875409; doi:10.1371/journal.pntd.0009114)
Supplement: S1 Text — (DOCX) [file pntd.0009114.s001.docx]

**Technical information on the Pharos micro prototype:** The thickness of the heating layer scales with $\sqrt{D\cdot t}$, where D is the thermal diffusivity of the reaction solution (on the order of $\cdot{1.5\cdot10}^{-7}m^{2}/s$) and t being the heating duration. By this approach, the heated mass of the wires and the surrounding liquid within the heating layer is a minute fraction ($\ll1\%)$ of the entire reaction volume. For the denaturation step, a 10 mF capacitator is loaded to 30-40 V, which is delivered for a sub-millisecond duration via a MOSFET (metal-oxide-semiconductor field-effect transistor; serving as a fast switch) to the wires that are heated to >90 °C to denature the amplicons on their surface. In order to keep the thickness of the heating layer as small as possible, a short heating duration is required, which can be achieved by applying a pulse at substantial peak power in the order of 1 kW to the wire array for a few hundred microseconds. Due to the short duration of each pulse, however, the average power consumption for the entire instrument is below 8 W (including the heat blocks, the fluorescence unit, the microcontroller and the pulsing).


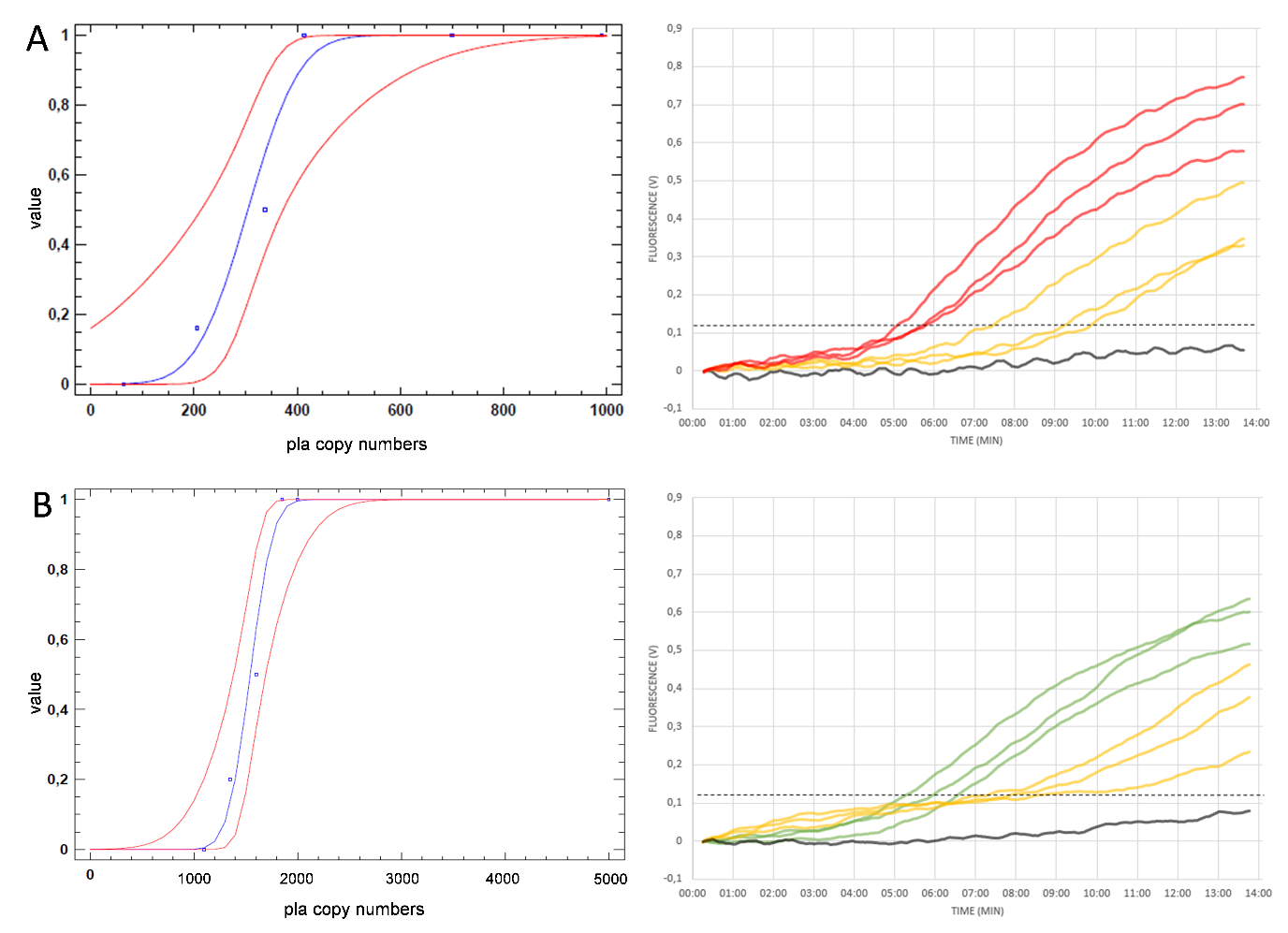


Fig. S1 Analytical limit of detection (A) Probit regression analysis (left) reveals a LOD (95%) of 434 pla copies per reaction for purified DNA. PCA results (right) of samples containing 1x10^4^ (red) copies per reaction and 413 (yellow) copies per reaction (negative control, black). (B) Probit regression analysis (left) reveals a LOD (95%) of 1824 pla copies per reaction. Considering that the EV76 strain used in this study was shown to contain 52 pPCP1 copies, this equals 35 cells per reaction. PCA results (right) of crude culture material containing 3.9x10^4^ (green) copies per reaction (≈750 cells) and 1800 (yellow) copies per reaction (≈34.6 cells).


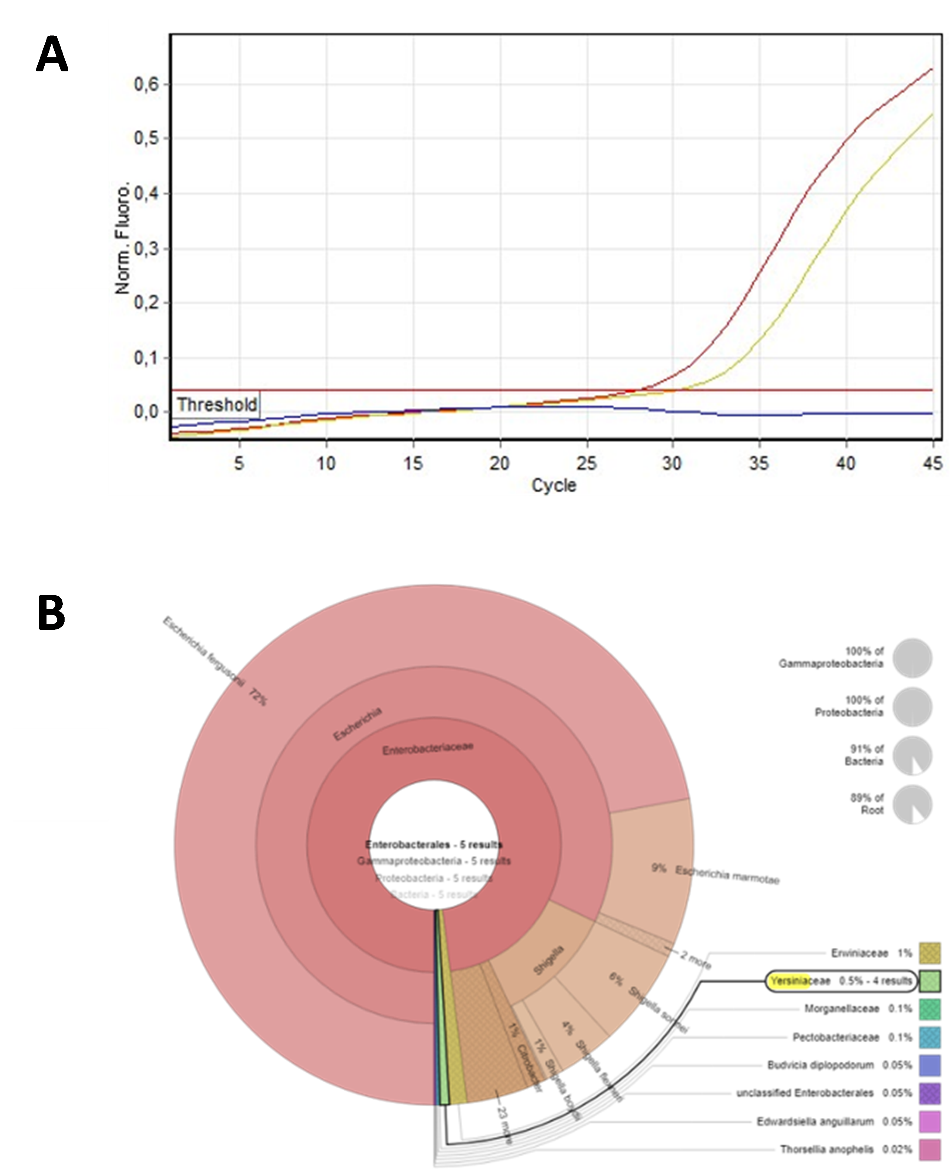


Fig. S2 Conventional qPCR and 16S sequencing confirm *Y. pestis* in field sample (A) Sample DNA was extracted and subjected to conventional pla specific qPCR in our stationary laboratory. Presence of *Y. pestis* was conformed (sample: yellow, positive control: red, negative control: blue) (B) Krona chart: Subsequent 16S sequencing of the sample revealed large amounts of contaminating bacteria (>95% *Enterobacteriacea*) and only little amounts (0.5%) of *Y. pestis*.

| Species | Strain |
| --- | --- |
| *Acinetobacter baumanii* | DSMZ 7324 |
| *Affenpocken Virus* | MSF-6 |
| *Bacillus anthracis* | Vollum |
| *Bacillus cereus* | ATCC 10987 |
| *Bacillus globigii* | DSMZ 7264 |
| *Bacillus thuringensis* | DSMZ 2046 |
| *Brucella abortus* | ATCC 23448 |
| *Burkholderia cepacia* | NCTC 10744 |
| *Burkholderia mallei* | Zagreb |
| *Burkholderia pseudomallei* | ATCC 23343 |
| *Burkholderia thailandensis* | DSMZ 13276 |
| *Campylobacter jejuni* | ATCC 29482 |
| *Candida albicans* | ATCC 36232 |
| *Chlamydophila pneumoniae* | ATCC 53592 |
| *Citrobacter freundii* | DSMZ 30039 |
| *Clostridium perfringens* | ATCC 12915 |
| *Clostridium sporogenes* | DSMZ 795 |
| *Coxiella burnetii* | Nine Mile |
| *Eikenella corrodens* | DSMZ 8340 |
| *Enterobacter aerogens* | DSMZ 30053 |
| *Enterobacter faecalis* | DSMZ 2570 |
| *Escherichia coli* | ATCC 11303 |
| *Francisella tularensis holarctica* | F 049 |
| *Haemophilus influenzae* | ATCC 10211 |
| *Klebsiella pneumoniae* | ATCC 13883 |
| *Legionella pneumophila* | NCTC 10332 |
| *Listeria monocytogenes* | DSMZ 12464 |
| *Moraxella catarrhalis* | DSMZ 9143 |
| *Mycobacterium tuberculosis* |  |
| *Neisseria meningitidis* | Patient isolate |
| *Proprionibacterium acnes* | DSMZ 1897 |
| *Proteus mirabilis* | DSMZ 788 |
| *Pseudomonas aeruginosa* | ATCC 10145 |
| *Salmonella enterica typhii* | 20-3267 |
| *Serratia marcescens* | DSMZ 1636 |
| *Shigella dysinteriae* | B476 |
| *Staphylococcus aureus* | DSMZ 19041 |
| *Staphylococcus epidermidis* | DSMZ 1798 |
| *Stenotrophomoas maltophilia* | ATCC 5131 |
| *Streptococcus pneumoniae* | DSMZ 20566 |
| *Streptococcus pyogenes* | DSMZ 20565 |
| *VACV* | VACV-0273 |
| *Vibrio cholerae* | ATCC 15748 |
| *Yersinia enterocolitica* | DSMZ 13030 |
| *Yersinia frederiksenii* | ATCC 33641 |
| *Yersinia pseudotuberculosis* | ATCC 29833 |

**Tab. S1** **No cross-reactivity was observed with any of the tested bacteria, confirming specificity of the pla assay** (ATCC: American Type Culture Collection; DSMZ German Collection of Microorganisms and Cultures; NCTC: National Collection of Type cultures)
