# Supplementary figures and images for: Pulse-Controlled Amplification–A new powerful tool for on-site diagnostics under resource limited conditions
Source: PLoS Negl Trop Dis. 2021 Jan 29;15(1):e0009114. doi: 10.1371/journal.pntd.0009114 (PMC7875409; doi:10.1371/journal.pntd.0009114)

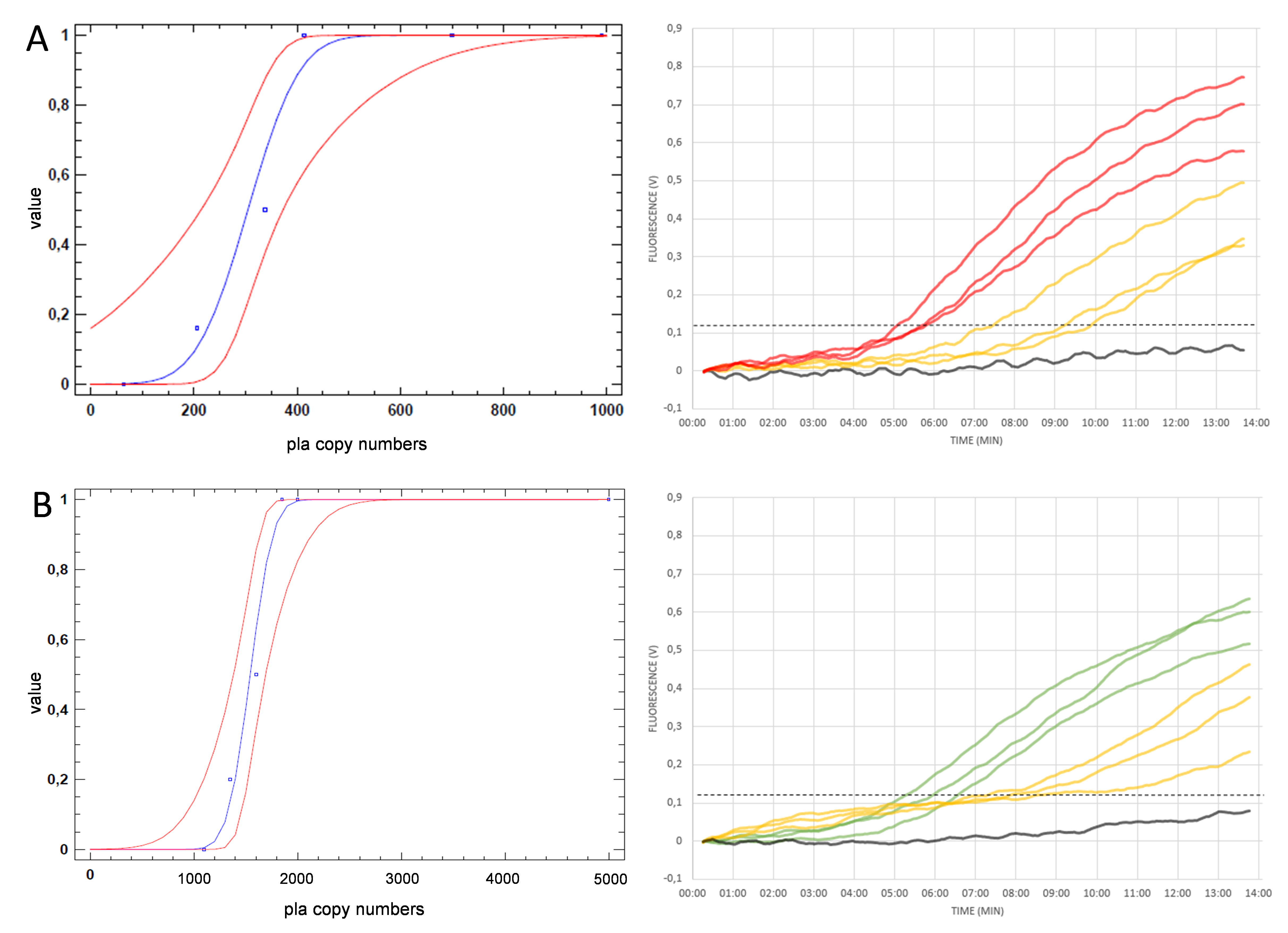

Supplement: S1 Fig — (A) Probit regression analysis (left) reveals a LOD (95%) of 434 pla copies per reaction for purified DNA. PCA results (right) of samples containing 1x10^4^ (red) copies per reaction and 413 (yellow) copies per reaction (negative control, black). (B) Probit regression analysis (left) reveals a LOD (95%) of 1824 pla copies per reaction. Considering that the EV76 strain used in this study was shown to contain 52 pPCP1 copies, this equals 35 cells per reaction. PCA results (right) of crude culture material containing 3.9x10^4^ (green) copies per reaction (≈750 cells) and 1800 (yellow) copies per reaction (≈34.6 cells). (TIF) [file pntd.0009114.s002.tif]

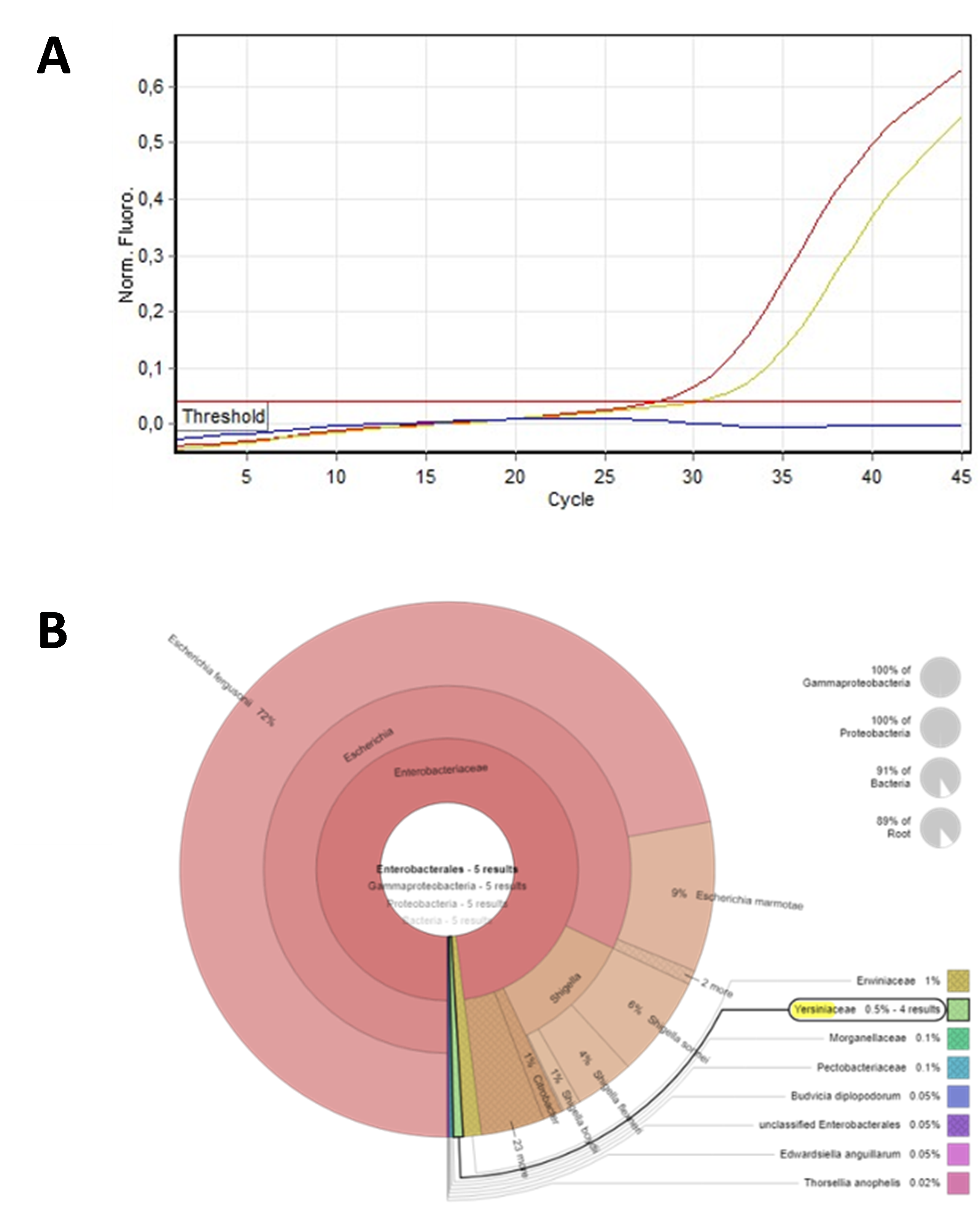

Supplement: S2 Fig — Conventional qPCR and 16S sequencing confirm Y. pestis in field sample (A) Sample DNA was extracted and subjected to conventional pla specific qPCR in our stationary laboratory. Presence of Y. pestis was conformed (sample: yellow, positive control: red, negative control: blue) (B) Krona chart: Subsequent 16S sequencing of the sample revealed large amounts of contaminating bacteria (>95% Enterobacteriacea) and only little amounts (0.5%) of Y. pestis. (TIF) [file pntd.0009114.s003.tif]
